# Two-dimensional and three-dimensional ultrasonography for pregnancy diagnosis and antenatal fetal development in Beetal goats

**DOI:** 10.14202/vetworld.2015.835-840

**Published:** 2015-07-09

**Authors:** Kailash Kumar, Ramesh Kumar Chandolia, Sarvan Kumar, Madan Pal, Kumar Sandeep

**Affiliations:** 1 Department of Veterinary Gynaecology & Obstetrics, College of Veterinary Science, Lala Lajpat Rai University of Veterinary and Animal Sciences, Hisar, Haryana, India; 2 Department of Veterinary Pathology, College of Veterinary Science, Lala Lajpat Rai University of Veterinary and Animal Sciences, Hisar, Haryana, India; 3 Department of Veterinary Surgery and Radiology, College of Veterinary Science, Lala Lajpat Rai University of Veterinary and Animal Sciences, Hisar, Haryana, India

**Keywords:** fetal development, goats, pregnancy, three-dimensional, two-dimensional, ultrasonography

## Abstract

**Aim:**

The objective of this study was to compare two-dimensional (2D) and three-dimensional (3D) study of the pregnant uterus and antenatal development of the fetus.

**Materials and Methods:**

2D and 3D ultrasound were performed from day 20 to 120 of gestation, twice in week from day 20 to 60 and once in week from day 60 to 120 of gestation on six goats. The ultrasonographic images were obtained using Toshiba, Nemio-XG (Japan) 3D ultrasound machine.

**Results:**

On the 20^th^ day of gestation, earliest diagnosis of pregnancy was done. First 3D ultrasonographic image of the conceptus, through transabdominal approach, was obtained on day 24. On 39^th^ day, clear pictures of conceptus, amniotic membrane, and umbilicus were seen. On 76^th^ day of gestation, internal organs of fetus *viz* heart, kidney, liver, urinary bladder, and stomach were seen both in 2D and 3D images. 3D imaging showed better details of uterine structures and internal organs of the fetus.

**Conclusions:**

Comparing 3D images with 2D images, it is concluded that 2D was better in visualizing fluid while 3D images were better to view details of attachment of fetus with endometrium.

## Introduction

Traditional methods for pregnancy diagnosis in small ruminants are palpation through the external abdomen and noting udder enlargement. In small size goats, X-ray can also be used for pregnancy diagnosis. However, these methods are applicable only in late pregnancy. Ultrasonography is quicker and less stressful and good tool for early pregnancy diagnosis. Two approaches of ultrasonography (transabdominal and transrectal) have been used with great accuracy as a means for pregnancy diagnosis and estimation of fetal numbers in goat [1,2]. Previously, several studies used two-dimensional (2D) ultrasonography to diagnose pregnancy and for measuring fetal dimension [2-5].

Diagnostic ultrasonography is a valuable alternating image system that can provide more accurate information about pregnancy and reproductive disorders in comparison to all traditional methods [6,7]. Early pregnancy diagnosis and fetal quantification through ultrasonography contribute to rationalize management and bring financial benefits to goat production [8,9]. Three-dimensional (3D) ultrasound is in use for the diagnosis of fetal abnormalities in human obstetrics and gynecology [10]. Several attempts to utilize 3D ultrasound in small animals’ practice have failed or led to a poor image quality due to problems with breathing artifacts by the slow acquisition time and software settings. This happened as the settings used for animals were the same as optimized for the human body [11].

The 3D technology offered advanced information about pregnancy status and birth prediction and improved the diagnostic confidence. By using standardized examination protocols, 3D ultrasound will allow a reduction in examination time by generating even more relevant information [11]. According to Cesarani *et al*. [12], the best 3D images were obtained from anatomical structures and pathologic conditions with a liquid content (i.e., bladder and gallbladder, cysts), or those adjacent to them (i.e., uterus and prostate). Therefore, the present study was designed to evaluate images of 3D ultrasonography over 2D ultrasonography for fetal development in the goat.

## Materials and Methods

### Ethical approval

The study was conducted after the approval of the Institutional Animal Ethics Committee.

### Animals

Six healthy pregnant goats of Beetal breed approximately 2 years of age having a history of normal reproductive performance were selected for the study. In all the animals, pregnancy was through natural mating. They were kept on grazing as well stall feeding.

### Ultrasonographic examination

Ultrasonography was conducted from day 20 after mating and continued till 120 days of gestation. The ultrasonography was conducted 2 times in a week from day 20 to day 60 and after this; the scanning was done once in a week. 2D and 3D ultrasonography were performed on each examination. No sedation was given to animals. The lower ventral and lateral abdomen area around teats of the goats were shaved, and the goats were positioned in lateral recumbency. There was no period of fasting before transrectal or transabdominal scannings.

### Instruments used

The ultrasound machine used for this study was 3D ultrasound machine (Nemio-XG: Toshiba, Japan) having 4D volumetric probe. During early pregnancy, 2D intra-operative probe of this machine having frequencies between 5 and 10 MHz was made stiff by fixing it on a 0.5” PVC pipe after splitting it and using adhesive tape. It was used for transrectal ultrasonography. Later on 2D convex transducer switchable between 3 and 6 MHz was used for 2D images. For the 3D images, the 3D mechanical volumetric probe having a frequency between 3 and 6 MHz was used. The stage of gestation at the time of examination was calculated from the date of mating.

### Statistical analysis

Differences at a p<5% (p<0.05) were considered to be statistically significant. All statistical analysis were performed using the SPSS (16.0) system for windows.

## Results

**Figure-1:**
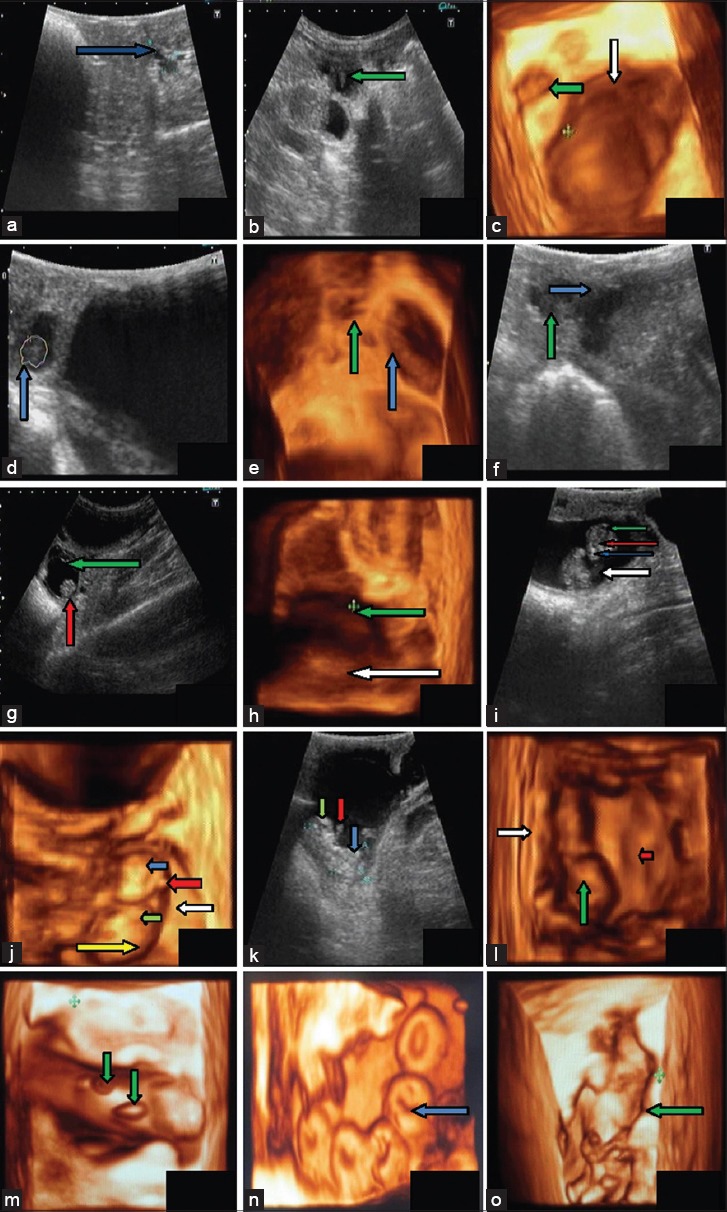
Typical images of fetal development from day 20 to day 60. (a). Trans-rectal ultra-sonogram of uterus of goats at day 20 of gestation with a 7.5 MHz trans-rectal transducer showed accumulation of anechoic fluid (blue arrow) in uterus, (b). Embryo proper was seen as elongated structure with tapering edge (indicating by green arrow) on one side of uterine lumen at day 24 of gestation, (c). Pregnant uterine horn at 24^th^ day of gestation towards cranial side of urinary bladder and tri-segmented fetus, (d). Trans-rectal ultra-sonogram of uterus of a goat at day 30 showing oblong shaped fetus (blue arrow) in uterine lumen towards cranial side of urinary bladder, (e). 3D ultra scan recorded at day 35 of pregnancy showing elongated conceptus (blue arrow) and urinary bladder (green arrow) of a goat, (f). Trans-rectal ultra-sonogram of uterus of a goat at day 35 showing accumulated large amount of fluid (blue arrow) at day 35 in uterine horns and presence of conceptus (green arrow), (g). Conceptus (blue arrow) showing attachment to one side of endometrium (red arrow) and hyperechoic amniotic membrane (green arrow) surrounding the fetus on day 39, (h). Image of fetus (green arrow) shows its presence in uterine lumen and endometrium (white arrow) was visible on day 39, (i). Head (green arrow), eyes (red arrow), nose (blue arrow) and fetal trunk (white arrow) are visible on day 42 of gestation., (j). Head (blue arrow), ear pinna (red arrow), forelimbs (green arrow), placenta (white arrow) and fetal trunk (yellow arrow) in 3D ultrasonic scan of fetus on day 42, (k). On day 48, eye ball (green arrow), forelimbs (red arrow) and fetal trunk (blue arrow) are seen. (l). 3D ultra-sonogram on day 48, using volumetric 4D trans-abdominal probe with 5.0 MHz transducer. Fetus (green arrow), endometrium (white arrow) and concave shaped depression (cotyledon) indicating by red arrow are seen in this image, (m). 3D scanning shows that fetus is surrounds by placenta and small raised structure was present on the placenta (shown by green arrow), (n). Full view of placentomes, which were ‘O shaped’ (blue arrow) with central depression surrounding a raised portion (day 54), (o). 3D (top view) scan of fetal body along with attachment of membrane and endometrium (green arrow) at day 60 of gestation.

In the current study, it was found that 2D and 3D ultrasonography were easily applicable without any significant risk to conduct study in pregnant uterus. Pregnancy was assessed as positive on day 20 of gestation by observing a small non-echogenic vesicle of 0.6 cm diameter with the help of per-rectal probe using 7.5 MHz frequency, however, on this day only uterus was enlarged and accumulation of fluid was seen, but there was no sign of conceptus (Figure-1a). 3D transabdominal scanning could not detect the fluid on day 20.

Embryo proper was first detected as a small echogenic structure on day 24 by both transrectal (7.5 MHz frequency) and transabdominal (4.2 MHz frequency) approaches. Conceptus was seen as an elongated structure with tapering edge on one side, and conceptus appeared attached to the endometrium toward other side (Figure-1b). In 3D images, accumulation of uterine fluid in one uterine horn was clearly seen toward the cranial side of urinary bladder and the conceptus was seen as tri-segmented structure, probably one for head, other for forelimb, and third one for hind limbs (Figure-1c). From the 3D view, it was possible to guess the location of head, forelimbs, and hindlimbs.

On 30^th^ day of pregnancy, conceptus was seen as oblong shaped in 2D scanning; similarly, in 3D scanning on this day, the conceptus was also seen as oblong shaped, showing attachment to side of membrane (Figure-1d). It was easily identified as anechoic structure with beating heart. The uterine layers were also clearly visible. The 3D image on this day also showed attachment of the conceptus to the endometrium. The earliest detection of placentome by transrectal sonography as circular echoic structure facing toward the fetus was made on 35^th^ day and in 2D scan, the amount of uterine fluid increased showing wavy margins of endometrium (Figure-1e). In 3D scanning, the elongated fetus was imaged surrounded by fluid and its attachment to the uterus was clearly seen (Figure-1f). Details of fetal attachment were clearly seen, and it appeared that 3D images were superior as compared to 2D on this day in matters of details of the uterus. The earliest detection of placentome by transrectal ultrasonography as circular echoic structure facing toward the fetus was made on day 35 and measured up to day 98 with the help of transabdominal approach. The placentome diameter was increased significantly during whole observation (p<0.05).

On the 39^th^ day, clear pictures of the fetus and hyperechoic amniotic membrane were seen (Figure-1g). Umbilicus was seen on day 39 of gestation. In 3D images, the fetus and endometrium were seen. Oblong shaped fetus was present in the uterine lumen (Figure-1h). On 42^nd^ day of pregnancy, the fetus was seen in the uterine lumen with easily identifiable head, ear-buds, folded forelimbs, and proper trunk in 2D scanning (Figure-1i). In 3D scanning, amazing view of fetus and details of its surrounding structures were seen (Figure-1j). Dorsal side of the fetus was surrounded by a thick band of the placenta from head to tail. Head, ear, forelimbs, umbilicus, and forelimbs were seen. Full details of the uterine structures including placenta and fetal attachments were seen.

On 48^th^ day of gestation, skull, rib cage, the spinal cord of the fetus, forelimbs, hind limbs, and other bony structures were seen in the 2D image (Figure-1k). To view the full fetus, the transducer was positioned between thighs and udder of the dam, however, with a slight pressure of transducer, fetus quickly changed its position. The fetus was mobile at this stage and by focusing on the fetus; rumination like the movement of mouth parts of the fetus was seen. Fetus and its body parts were identifiable with the help of 3D scanning on this day. The details of placenta toward limb and thickness of endometrium on one side of the fetus were seen while a broadsheet of placenta was seen on other side of the fetus. Depression of concave shaped cotyledons was also seen on the flat placenta (Figure-1l).

On the 54^th^ day of gestation, placenta, fetus, and the endometrium were clearly visible. In 2D scan, fetus showed movements in the fetal fluid. Fetus enlarged in size, and it was difficult to get full fetus in one frame. Therefore, the fetus was imaged in parts. The head of fetus and rest of body were embedded in anechoic fetal fluid and to image the fetal head, transducer needed to focus on head area for quite some time. 3D imaging on this day showed details of the placenta. The structures present on the placenta, surrounding the fetus, were of two kinds. One was elliptical, and other one was circular, and both were raised from placenta (Figure-1m). Details of placental attachment to the endometrium were also seen. The endometrium was contracted on this structure in wavy form. This kind of details was not seen in 2D imaging. Surface view of 3D scanning provided a full view of placentomes, which were ‘O’ shaped with central depression surrounding a raised portion (Figure-1n).

At the 60^th^ day of gestation in 2D scanning, it was found difficult to get full fetus in one scan, therefore, fetal trunk area or head was focused at one time. 3D ultra-scan on this day, as reported in 2D, the body parts were in focus and not the full fetus. Detailed structures surrounding head were seen depicting protection of the fetal head (Figure-1o). Further in this figure, surface top-view of the fetus showed fetal limb, abdomen, hind limbs, and part of the head. It appeared that the attachment of the fetal membrane to endometrium was not simple, but complicated. There were elongated and round projections on membranes that extended from endometrium over to the head. The endometrium was also wavy, leaving spaces in between.

**Figure-2:**
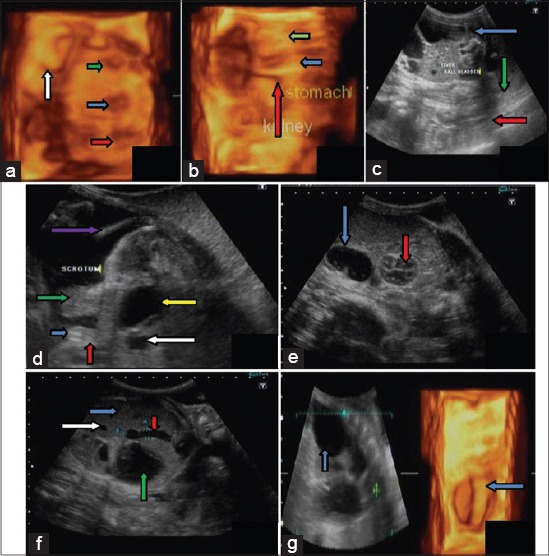
Fetal development from day 60 to day 120: (a) On day 76 of gestation, heart (green arrow), stomach (blue arrow) and urinary bladder (red arrow) showing in 3D ultra sonogram. Cotyledon are seen as ‘C’ shaped (white arrow), (b) Liver (blue arrow), Kidney (red arrow) and stomach (green arrow at day 98 of gestation (c) Liver (green arrow), gall bladder (red arrow) and parts of stomach (blue arrow) at day 82 of gestation in 2D image, (d) On day 105 blobbed hyper echoic genital tubercle (blue arrow), pendulous scrotum (green arrow), umbilicus (red arrow), umbilical well (white arrow), tail (violet arrow) and urinary bladder (yellow arrow), (e) Stomach with echogenic particles (blue arrow) and kidney (red arrow) is visible in 2D ultra-sonogram at day 120 of pregnancy, (f) Liver (blue arrow), gall bladder (red arrow), stomach (green arrow) and hepatic vessel in 2D ultra sonogram at day 120 of pregnancy, (g) Blobbed stomach (blue arrow) of fetus in 3D ultra sonogram at day 120 of gestation.

Both 2D and 3D image showed greater details of organs on the 76^th^ day of gestation. On this day internal organs *viz* heart, kidney, urinary bladder, stomach, and liver of fetus were easily identifiable (Figure-2a). In the present observations, there was a rapid growth of internal organs around and after day 76 of gestation. The scrotum in the male fetus was identified on the 82^nd^ day of gestation. In earlier studies, genital tubercle has been reported by various investigators.

Between 110 and 120 days of gestation complete details of the fetal stomach, heart, liver, gall bladder, kidney, and urinary bladder were seen in both 2D and 3D ultrasonographic images (Figure-2b-g).

## Discussion

Ultrasonography is an important tool for early pregnancy diagnosis. The stage of gestation at the time of examination was calculated from the date of mating. In the present study, pregnancy was observed as a small non-echogenic vesicle of 0.6 cm diameter on day 20 of gestation with the help of per-rectal probe with 7.5 MHz frequency. This is in agreement with Padilla-Rivas *et al*. [13]. The investigators reported a small non-echogenic vesicle of about 1 cm in diameter in the uterine lumen by day 21. Martinez *et al*. [14] detected an embryonic vesicle on day 18 having 4.1 mm size. Similarly, Medan *et al*. [15] reported the appearance of a circular or elongated gestational sac in the uterine lumen on days 20.2±0.6 of pregnancy. It is concluded that the early detection of pregnancy in goat is possible around day 20.

In the present study, the accuracy of ultrasound was 100% for detecting pregnant and non-pregnant cases. Almost all previous investigators have used transabdominal ultrasonography for pregnancy detection in goat except Martinez *et al*. [14] used transrectal transducer for pregnancy diagnosis. This might be possible by the use of a different frequency of the transducer and advanced 3D ultrasound machine.

In 3D images, the conceptus was seen as tri-segmented structure, probably one for head, other for forelimb, and third one for hind limbs. From the 3D view, it was possible to guess the location of head, forelimbs, and hindlimbs. The segmented appearance of conceptus has also been reported in canine [16,17]. Therefore, use of 3D in the present study appeared more useful than 2D for the better details of the conceptus in the goat. This transabdominal detection in the present study was 4 days earlier than observation of Martinez *et al*. [14]. Heartbeats were detected on day 24 in the present study, which is in agreement with the study of Medan *et al*. [15], Martinez *et al*. [14] and with most of other authors who reported heart beats around this time of pregnancy in goats [13,18].

On the 30^th^ day of pregnancy, the fetus was surrounded by anechoic fluid and its location was toward the cranial side of urinary bladder, which is in agreement with the study conducted by Martinez *et al*. [14]. The earliest detection of placentome by transrectal ultrasonography as circular echoic structure facing toward the fetus was made on day 35 and measured up to day 98 with the help of transabdominal approach. Other investigators reported that placentomes appear first as small echogenic densities in the wall of the uterus at days 26-28 of gestation [19-21]. In some studies, placentomes were recognized clearly at 7 weeks of gestation or at day 38 of gestation [13]. Umbilicus was seen on day 39 of gestation, which was 9 days later than seen by Karen *et al*. [5] in Egyptian goats. These investigators reported the presence of umbilical cord on the 30^th^ day of gestation. It might be due to the difference in the breed. In 3D images, the fetus and endometrium were seen. On 42^nd^ day of pregnancy, full shape of the fetus was seen, and its body parts were easily identifiable. In 3D scanning, amazing view of fetus and details of its surrounding structures were seen. There is no parallel report of 3D ultrasonography of goat pregnancy.

On 48^th^ day of gestation, skull, rib cage, the spinal cord of fetus, forelimbs, hind limbs, and other bony structures were seen in 2D image which was 1-week earlier than Suguna *et al*. [2]. Medan *et al*. [15] also reported that skeletal structures were obvious at 2 months of pregnancy, almost 12 days later that present observations. The fetus was mobile at this stage and by focusing on the fetus; rumination like the movement of mouth parts of the fetus was seen. Chandolia *et al*. [22] also reported movement of body part of the fetus on day 38 after conception in the goat.

On 54^th^ day of gestation, surface view of 3D scanning provided a full view of placentomes, which were ‘O’ shaped with central depression surrounding a raised portion. Similar to our findings of 2D images, Lee *et al*. [9] had reported that the placentome increases in size and appeared as a ‘C’ or ‘O’ shaped. The 2^nd^ month of pregnancy has been reported to be the best period for imaging placentomes [2].

On 76^th^ day of gestation, both 2D and 3D images showed greater details of organs *viz* heart, kidney, urinary bladder, stomach, and liver of the fetus. There is no parallel study, reported on 3D ultrasonography. Similarly, no parallel study is available in goat regarding the development of fetal organs in utero (antenatal) using B-mode ultrasonography. Matsas [23] had also reported that the fetal skeleton grows rapidly between second and 3^rd^ month of gestation.

The scrotum in the male fetus was identified on the 82^nd^ day of gestation. In earlier studies, genital tubercle has been reported by various investigators. Ramphal [24] has reported genital tubercle in ram at day 53 of gestation and scrotum on day 90 of gestation. Lack of more reports on this area could be due to less work on advanced pregnancy. Yotov *et al*. [25] reported that the fetal sex can be determined best in sagittal or cross-sectional position in buffaloes.

Between 110 and 120 days of gestation complete details of fetal stomach, heart, liver, gall bladder, kidney, and urinary bladder were seen in both 2D and 3D ultrasonographic images but there is no previous parallel study in this area.

## Conclusion

For early pregnancy diagnosis, the transrectal approach was better than the transabdominal approach. Early pregnancy could be detected at 20 days of gestation with 7.5 MHz transducer. Fetal development in great details, a particularly segmented form of the embryo could be observed in 3D ultrasonography on day 24. Conceptus changed its shape from 24 to 42 days of gestation, and full identifiable conceptus took its shape on day 42. 3D ultrasonographic procedure gave more details of the fetus, placenta, and attachment of the fetus to the endometrium, and this might be used as a diagnostic tool in future veterinary obstetrics. Images of internal organs of the fetus were viewed in details both in 2D and 3D images, which might be used as a future guide for antenatal assessment of normal or abnormal conceptus.

## Authors’ Contributions

KK and RKC have designed the study and planned the research experiments. KK performed the research experiments. RKC supervised the research. SK, MP, and SK help in conducting experiment. All authors read and approved the final manuscript.
